# Reducing CSF complications by a recycled Hadad’s flap combined with autologous mucosa in secondary endoscope transsphenoidal surgery

**DOI:** 10.3389/fonc.2023.1224804

**Published:** 2023-08-02

**Authors:** Runfeng Wang, Gaoyang Zhou, Jin Wang, Bo Ma, Ping Wang, Guodong Gao, Shukai Sun, Zhiguo Zhang

**Affiliations:** Department of Neurosurgery, Tangdu Hospital, The Air Force Military Medical University, Xi’an, Shaanxi, China

**Keywords:** repeated transsphenoidal surgery, Hadad-Bassagasteguy flap, CSF complications, skull base surgery, transsphenoid approach

## Abstract

**Background:**

Transsphenoidal secondary operations are a minority but not a rare occurrence. How to viably prevent cerebral fluid (CSF)-related complications and confine surgery-caused injury in secondary surgery as minimally as possible is a huge challenge. This article shares our solution of recycling a prior Hadad-Bassagasteguy flap (HBF) along with a using small piece of free autologous mucosa to reconstruct the skull base.

**Methods:**

Of 69 patients, fitted criteria were assigned into 2 different groups: a recycled HBF incorporated with an autologous free mucosa and a recycled HBF incorporated with an artificial dura to rebuild the skull base in secondary transsphenoidal surgery. The postoperative morbidities of pseudomeningocele, CSF leakage and meningitis were recorded and analyzed.

**Results:**

A recycled HBF incorporated with an autologous mucosa is capable of reducing CSF complications compared to that of the matched group, particularly decreasing the morbidity of meningitis in secondary transsphenoidal surgery. Diabetes mellitus, craniopharyngioma, chordoma and the utilization of artificial dura were independent risk factors for CSF complications in secondary transsphenoidal surgery through univariate and multivariate logistic regression. In addition, diabetes mellitus and artificial dura are more likely to induce CSF leakage and meningitis. Patients suffering from craniopharyngioma are more susceptible to meningitis. Chordoma indiscriminately increased the risk of each CSF complication.

**Conclusion:**

A recycled HBF incorporated with an autologous mucosa is reliable for reconstructing the skull base in secondary transsphenoidal surgery, especially for patients simultaneously suffering from diabetes mellitus and central skull base tumors.

## Background

Over the past 2 decades, along with developments in endoscopic equipment and anatomy exploration, neurosurgery has accomplished great success in operative treatment for skull base tumors. Lesions located in the cerebral ventral region only used to be touched through a complicated transcranial approach, which might induce extra damage and prolonged hospitalization. Now, by using an endoscope with an expanded endoscopic endonasal approach (EEA), neurosurgeons can not only easily explore the ventral cerebrum region but are also capable of resecting tumors with minimal interference to other adjacent structures. As a coin holds two sides, the disadvantage of the expanded EEA is as obvious as its advantage, which is the induction of cerebrospinal fluid (CSF)-related complications. Due to the EEA’s special trajectory and the location of the skull base lesion, isolation between the nasal cavity and the intracranial space is a vital procedure for decreasing the morbidity of CSF complications after resection. In the past, the rate of postoperative CSF complications was >20% until the introduction of the Hadad-Bassagasteguy flap (HBF) for expanded EEA (1). Currently, it has dramatically decreased to only <5% (2–6), which is comparable with that of the traditional transcranial approach (7). However, along with the ubiquitous utilization of expanded EEA worldwide, for some selected but not rare patients, CSF complications are still a huge challenge. This challenge might be life-threatening for certain patients who have to undergo a secondary EEA surgery. In prior surgery, neurosurgeons initially harvested an HBF to prevent anticipated CSF leakages. This HBF occupies the ipsilateral nasal septal artery (NSA) as its blood pedicle. Meanwhile, to fully visualize vital anatomical landmarks located at the posterior wall of the sphenoid sinus, sphenoidectomy and nasal septectomy are usually performed to gain binaural and bimanual access. Such a maneuver always interrupts the contralateral NSA, which sacrifices the capability of employing the contralateral septal mucosa as a rescued HBF for secondary surgery (8). Given the essential role of the vascularized flap for the reconstruction of the skull base, to obtain a vascularized flap again, Kassam et al. employed a pedicled inferior turbinate flap (IPTF) to restore the boundaries between the nasal cavity and subarachnoid space (9). Carnevale et al. introduced a method with a pedicled middle turbinate flap (MPTF) to cover the skull base defect (10). In addition, a temporoparietal facia flap was employed to restore dural defects after expanded EEA by Gardner (11). Surely, these kinds of methods are good substitutes for reconstruction when an HBF is absent in repeated EEA surgery; however, when considering this consensus that improving patients’ postoperative life quality and minimizing surgery-related injury as much as possible, they all hold some drawbacks. After harvesting the MTPF and ITPF, severe crusting and nasal pain frequently arise postoperatively due to a more denuded bone surface. Furthermore, the sizes of the MTPF and ITPF are smaller than that of the HBF. Due to their irregular shape of inferior and middle turbinates, MTPF and ITPF are more fragile and can be easier to rupture when elevating from the bone surface. Regarding the temporoparietal facia flap, an extra hemicoronal incision is needed, and a series of destructive maneuvers are performed to transfer it into the nasal cavity. Thus, regarding secondary EEA, how to properly confront CSF complications and maintain patients’ quality of life is a dilemma. Upon this delicate issue, recycling a prior HBF is hardly an unreasonable option. When elevating a prior HBF in secondary surgery, however, it is always inevitably ruptured due to a tight adhesion between the flap and the bone or dura, which leads to a shortened flap that is not capable of fully covering the dura defect. Thus, a small autologous or artificial “patch” is needed to compensate for its shortened size. Here, we are the first to introduce our clinical experience of comprising a prior HBF with free autologous nasal septal mucosa to rebuild the skull base and share our results about the independent risk factors for CSF complications in secondary EEA surgery.

## Methods

### Patient population

With the approval of our institutional review board, we retrospectively reviewed the related medical files of patients treated with EEA surgery at the Second Affiliate Hospital of the Air Force Medical University of China between February 2017 and February 2023. The enrolled patients met all the inclusion criteria, which were as follows: patients with tumor recurrence, suffered related symptoms and local neural disorders needed to be treated with secondary EEA surgery, availability of the pedicle of prior HBF confirmed by intraoperative Doppler probe test (12), interruption of the contralateral NSA by prior EEA surgery, and confirmed intraoperative CSF leak. Patients were excluded if they met any of the exclusion criteria listed as follows: the pedicle of prior HBF was unavailable, as confirmed by intraoperative Doppler probe test; a prior HBF was difficult to harvest in the secondary EEA surgery due to tight adhesions; no CSF leak occurred during secondary EEA surgery; and patients once received radiotherapy. Different duraplasty “patch” were randomly selected during operation. Based on the different selections of this “patch”, patients were assigned into 2 subgroups: a group reconstructed with prior HBF incorporated with an autologous nasal septal mucosa and a group reconstructed with prior HBF incorporated with artificial dura. All patients were clinically followed for at least 3 months. The postoperative presentation of CSF leak, pseudomeningocele, meningitis and nasal morbidity were recorded during hospitalization and the follow-up period. Typical symptoms (clear fluid dropping/orthostatic headache/stiff neck) combined with high-resolution CT/MRI tests were used to diagnose CSF leakage postoperatively. Postoperative pseudomeningocele was confirmed by T_2_-weighted MRI. Infectious organisms in the CSF were confirmed by bacterial cultivation and Gram staining when patients presented with any of the following symptoms: fever (>38), headache, stiff neck, meningeal signs or irritability. The Sinonasal Outcome Test-22 (SNOT-22) was utilized before and 1, 2, and 3 months after the secondary EEA surgery to assess the patients’ nasal morbidity (13).

### Surgical technique

**Figure 1:**
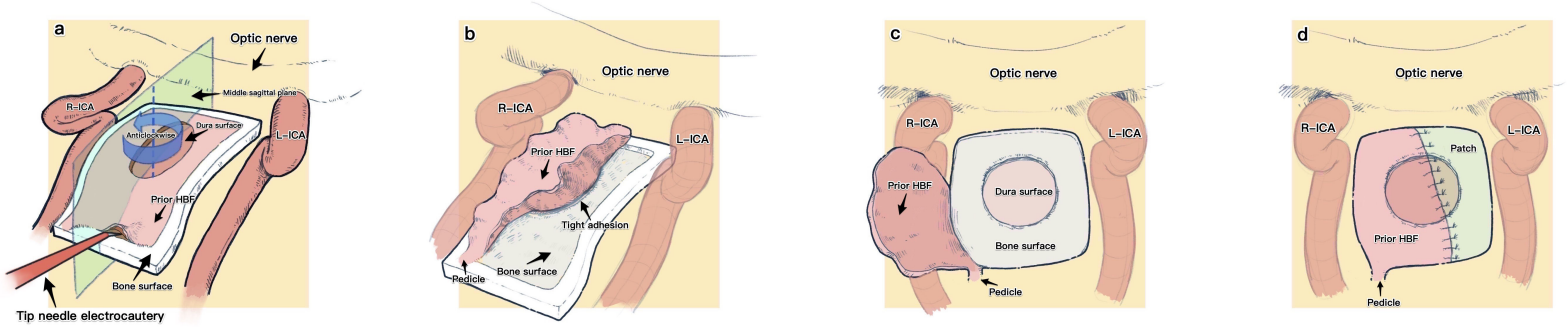
Schematic figure shows our surgical details of harvesting and resetting a prior HBF.

To obtain neat access through the nasal cavity, a solution of 1% lidocaine mixed with 1/100000 epinephrine was employed to decongest the mucosa for preparation. Due to enlarged manipulations contributed by the prior surgery, there was no need to out-fracture the middle and inferior turbinate to gain more space in the secondary surgery. The endoscope was inserted into the bilateral nasal tract to check the size of the residual nasal septa mucosa and then to assess the blood flow of the prior HBF pedicle and contralateral NSA with a Doppler probe. A prior HBF usually covered an area similar to a concentric circle shape, and the dura surface was encircled by the bone surface. Considering that elevating a prior HBF from a bone surface was relatively safe compared to elevating it from a dural surface, we always elevated it initially from the bone surface and then from the dural surface. First, we always observed the whole range of a prior HBF. Subsequently, at the most protuberance of residual sphenoid bone in the middle sagittal plane of the skull, needle tip electrocautery was used to expose the interface between the mucosa and bone. The flap was gradually elevated from the bone surface along the boundaries exposed before and then in an anticlockwise manner to further elevate it from other bone surfaces (**Figure 1A**). After the prior HBF was freely detached from all the bone surface, a tight adhesion could always be observed between the flap and the dura (**Figure 1B**). Careful maneuvers should be performed when this adhesion is detached. Such tight adhesions at this area always cause turbulent venous bleeding and lead to a ruptured and shortened prior HBF, which inevitably needs an extra “patch (autologous/artificial)” to compensate for the short size. After the extirpative phase was over, we usually reset the prior HBF upon the dura defect by repeated comparison to precisely estimate the size of a customized “patch”. According to the result, a small piece of mucosa graft was obtained from the residual nasal septa or a small artificial dura was trimmed as an extra “patch” that was directly sutured or bonded to the prior HBF (**Figures 1C, D**). Subsequently, we rotated this extended prior HBF to satisfactorily seal the entire dural defect.

### Statistical analysis

SPSS Statistics 20.0 (IBM) was employed to analyze all data. The mean and standard deviation are used for normally distributed data. The median is used for nonnormally distributed data. Categorical data are presented as frequencies or percentages. Univariate and multivariate logistic regression models were used to explore independent risk factors. Multinomial logistic regression was employed to determine the correlation between those independent risk factors and postoperative CSF complications. Variables were compared between 2 groups by Student’s t tests, chi-square tests or continuity correction tests. Differences with P values < 0.05 were defined as statistically significant.

## Results

After scrutinizing the related medical files, 69 patients who underwent secondary EEA surgery were enrolled in this study. Approximately 37 male and 32 female patients were included in this cohort, whose mean age was *50.8710.90* years. Based on the “patch” option, the patients were assigned into 2 groups: prior HBF incorporated with autologous mucosa (*n=41*) and prior HBF incorporated with artificial dura (*n=28*). The lesions involved in this study included meningioma (*n=5*), craniopharyngioma (*n=5*), chordoma (*n=6*) and pituitary adenoma (*n=53*). There was no significant difference between these 2 groups regarding age (*49.509.54 years, 51.8011.77 years, p=0.392*), sex (*p=0.628)*, BMI (*p=0.323*), diabetes mellitus (*p=0.933*), intraoperative communication of the 3^rd^ ventricle (*p=0.315)*, pathological diagnosis (*p>0.38*), or the rate of total resection (*p=0.698*). There were no postoperative mortalities (death in the immediate postoperative course or within 1 month after operations). All patients were clinically followed until death or loss of contact. The mean follow-up was 30.615.8 months. Detailed information about the patients is listed in **Table 1**.

**Table 1:** Baseline characteristics of 2 groups which duraplasty with different patch.

Variables	Total n=69	Graft Material		p value
		Artificial n=28	Autologous n=41	
Gender				
Female	32 (46.4)	12 (42.9)	20 (48.8)	0.628
Male	37 (53.6)	16 (57.1)	21 (51.2)
Age	50.87±10.90	49.50±9.54	51.80±11.77	0.392
BMI	25.90±1.6	25.67±1.78	26.06±1.46	0.323
Diabetes Mellitus				
Yes	12 (17.4)	5 (17.9)	7 (17.1)	0.933
No	57 (82.6)	23 (82.1)	34 (82.9)
Pathology diagnosis				
Pituitary adenoma	53 (76.8)	20 (71.4)	33 (80.5)	0.381
Craniopharyngioma	5 (7.2)	3 (10.7)	2 (4.9)	0.656
Meningioma	5 (7.2)	3 (10.7)	2 (4.9)	0.656
Chordoma	6 (8.7)	2 (7.1)	4 (9.8)	>0.99
Gross Total Resection				
Yes	59 (85.5)	25 (89.3)	34 (82.9)	0.698
No	10 (14.5)	3 (10.7)	7 (17.1)
Intraoperative Perforation Of the 3rd Ventricle				
Yes	10 (14.5)	6 (21.4)	4 (9.8)	0.315
No	59 (85.5)	22 (78.6)	37 (90.2)
Complications				
Yes	14 (20.3)	10 (35.7)	4 (9.8)	0.008
No	55 (79.7)	18 (64.3)	37 (90.2)
Pseudomeningocele				
Yes	3 (4.3)	1 (3.6)	2 (4.9)	>0.99
No	66 (95.7)	27 (96.4)	39 (95.1)
CSF leak				
Yes	4 (5.8)	3 (10.7)	1 (2.4)	0.358
No	65 (94.2)	25 (89.3)	40 (97.6)
Meningitis				
Yes	7 (10.1)	6 (21.4)	1 (2.4)	0.031
No	62 (89.9)	22 (78.6)	40 (97.6)

As shown in **Table 1**, in the artificial patch group, there was 1 patient with a pseudomeningocele (*3.6%*), 3 patients with a CSF leak (*10.7%*) and 6 patients with meningitis (*21.4%*). In the autologous patch group, there were 2 patients with a pseudomeningocele (*4.9%*), 1 patient with a CSF leak (*2.4%*) and 1 patient with meningitis (*2.4%*). A significant difference was observed between these 2 groups regarding postoperative CSF-related complications (*p=0.008*). In particular, the employment of autologous patches dramatically reduced the incidence of postoperative meningitis (*p=0.031*).

**Figure 2:**
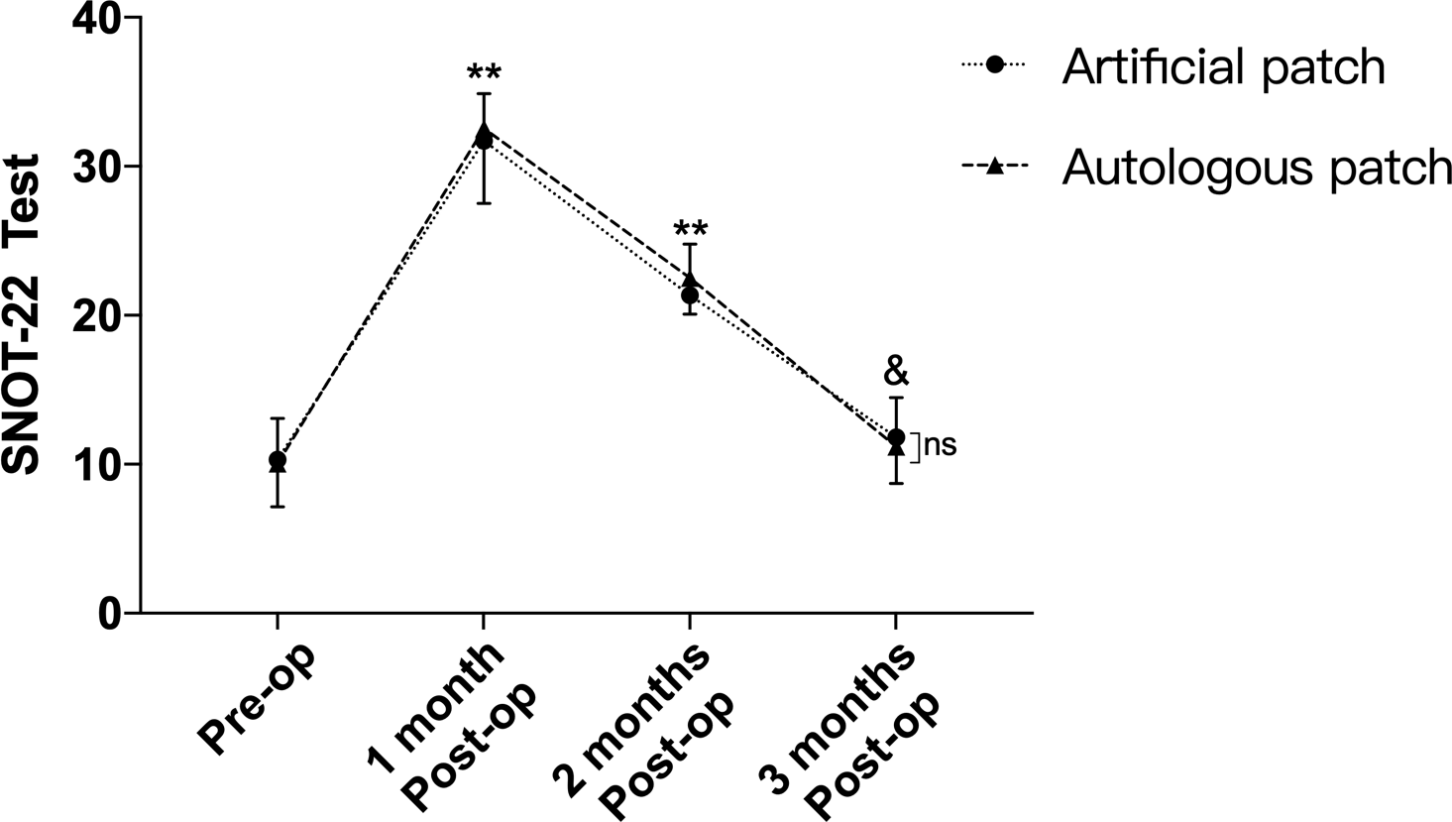
Though obvious differences of SNOT-22 test were observed between pre-operation and 1,2-month post-operation, no difference was confirmed between pre-operation and 3-month after operation. Meanwhile, there was no difference between 2 groups of SNOT-22 test regarding to pre-operation and 1, 2, 3-month after the secondary EEA operation. ^**^p<0.001, ^&^p>0.05, compared with pre-operation by two-way repeated measures ANOVA. ns, no significant.

As shown in **Figure 2** and **Table 2**, according to the SNOT-22 test, there was no significant difference between the 2 groups preoperatively (*p=0.698*). The 1-month and 2-month postoperative SNOT-22 test scores were obviously increased compared with the preoperative scores (*p<0.001*). However, 3 months after the secondary EEA surgery, the score of the SNOT-22 test gradually returned to the preoperative level (*p=0.056, p=0.08*). Meanwhile, no significant difference was observed between the 2 groups regarding the SNOT-22 test at 1 month, 2 months and 3 months postoperatively (*p=0.409, p=0.131, p=0.318*).

**Table 2:** Preoperative and postoperative SNOT-22 tests of 2 groups.

Sinonasal Outcome Test-22	Preoperative	1 Month Postoperative	^&^p Value	2 Month Postoperative	^&^p Value	3 Month Postoperative	^&^p Value
Artificial Mucosa	10.32±2.76	31.71±3.18	<0.001	21.36±3.43	<0.001	11.82±2.65	0.056
Autologous graft	10.05±2.90	32.54±5.03	<0.001	22.51±2.43	<0.001	11.20±2.46	0.08
^#^p Value	0.698	0.409	–	0.131	–	0.318	–

& indicates comparations between the preoperative data and each of the 1, 2, 3-month postoperative data regarding to the SNOT-22 tests.

# indicates comparations of 2 groups at pre-operation and 1, 2, 3-month after operation regarding to the SNOT-22 tests.

To further evaluate the effectiveness of the autologous patch and detect related independent risk factors for postoperative CSF complications, multiple variables, such as age, sex, BMI, pathology type, diabetes mellitus, gross total resection ratio (GTR), duraplasty material type and intraoperative perforation of the 3^rd^ ventricle, were included in our study according to the primary literature and clinical experience (14–16). Univariate and multivariate logistic regression were used to analyze multiple variables. Variables with p<0.05 detected by univariate logistic regression were included into the multivariate logistic regression. In our series, as shown in **Table 3**, artificial patches (*p=0.013*), diabetes mellitus (*p=0.011*), craniopharyngioma (*p=0.011*), and chordoma (*p=0.043*) were confirmed to be independent risk factors through the univariate and multivariate logistic regression. Age (*p=0.716*), sex (*p=0.768*), BMI (*p=0.621*), GTR ratio (*p=0.414*) and intraoperative perforation of the 3^rd^ ventricle (*p=0.414*) were not independent risk factors for secondary EEA surgery.

**Table 3:** Univariate and multivariate logistic regression of independent risk factors for CSF complications in secondary transsphenoidal surgery.

Variables	Crude	Adjusted
	OR (95% CI) p Value	OR(95% CI) p Value
Gender	1.195 (0.366-3.904) 0.768	
Age	0.990 (0.937-1.045) 0.716	
BMI	1.099 (0.755-1.601) 0.621	
Diabetes Mellitus		
Yes	17 (3.915-73.817) <0.001	21.904 (2.050-234.055) 0.011
No	Ref	
Pathology diagnosis		
Pituitary adenoma	Ref	
Chordoma	18.375 (2.344-144.043) 0.006	14.680 (1.083-199.044) 0.043
Craniopharyngioma	24.500 (3.383-177.423) 0.002	28.468 (2.144-378.067) 0.011
Meningioma	18.375 (2.344-144.043) 0.006	8.662 (0.481-155.943) 0.143
Gross Total Resection		
Yes	0.535 (0.119-2.403) 0.414	
No	Ref	
Intraoperative Perforation Of the 3rd Ventricle		
Yes	1.870 (0.416-8.405) 0.414	
No	Ref	
Duraplasty Material		
Artificial Graft	5.139 (1.416-18.651) 0.013	26.395 (2.003-347.891) 0.013
Autologous Mucosa	Ref	

To further assess the influence of the independent risk factors above on each of the CSF complications (pseudomeningocele, CSF leak, meningitis), multinomial logistic regression was employed to detect the association among them. As shown in **Table 4**, diabetes mellitus and the utilization of artificial patches were more likely to induce CSF leak (*p=0.008, p=0.05*) and meningitis (*p=0.02, p=0.02*) than pseudomeningocele (*p=0.066, p=0.466*). The patients suffering from craniopharyngioma were more susceptible to meningitis (*p=0.038*) than CSF leak (*p=0.112*) and pseudomeningocele (*p=0.196*). Chordoma indiscriminately increased the risk for all 3 kinds of postoperative CSF complications (*p=0.1, p=0.157, p=0.254*).

**Table 4:** Multinomial logistic regression of risk factors for postoperative CSF complications.

Variable	Total	No Complications ^ref^	Pseudomeningocele	p Value	CSF leak	p Value	Meningitis	p Value
Duraplasty Material								
Autologous Mucosa ^ref^	41	37	2 (4.9%)		1 (2.4%)		1 (2.4%)	
Artificial Graft	28	18	1 (3.6%)	0.466	3 (10.7%)	0.05	6 (21.4%)	0.02
Diabetes Mellitus								
No ^ref^	57	51	1 (1.8%)		1 (1.8%)		4 (7%)	
Yes	12	4	2 (16.7%)	0.066	3 (25%)	0.008	3 (25%)	0.02
Pathology Diagnosis								
Others ^ref^	58	51	1 (1.7%)		2 (3.4%)		4 (6.9%)	
Chordoma	6	3	1 (16.7%)	0.1	1 (16.7%)	0.157	1 (16.7%)	0.254
Craniopharyngioma	5	1	1 (20%)	0.196	1 (20%)	0.112	2 (40%)	0.038

## Discussion

In recent years, an increasing number of neurosurgeons have chosen EEA as their priority when confronting brain ventral tumors. Regarding related postoperative CSF complications, specific techniques and clinical data have been elucidated. However, to the best of our knowledge, few studies have considered the needs of patients who might experience a secondary EEA operation. These patients are minority but are not rare. During a secondary EEA surgery, a more extensive bone window was sometimes performed to gain a better view. Meanwhile, due to the lack of an accessible vascularized graft, a water-tight closure might not be accomplished when compared to that of the initial surgery. Thus, exploring an accessible, effective and safe substitute for HBF in secondary EEA surgery is a crucial issue. In the present study, we aimed to introduce a method that could not only confine the damage as much as possible but also hold considerable effectiveness in secondary EEA surgery. In our view, recycling the prior HBF is a reasonable option. According to our results, a recycled HBF incorporated with autologous free mucosa was effective in preventing CSF complications for secondary EEA surgery, which was comparable to that of the initial EEA surgery that rebuilt the skull base with a whole HBF (14–17). Moreover, we consecutively recorded the SNOT-22 scores before and 1 month, 2 months, and 3 months after the operation for the 2 groups. Although the SNOT-22 score was distinctly increased 1 month and 2 months postoperatively compared to those pre-operation, no significant difference was observed between pre-operation and 3 months postoperation for each group, and no significant difference was confirmed between the 2 groups at the 1-month, 2-month and 3-month postoperative SNOT-22 tests. Thus, our solution did not aggravate patients’ sinonasal quality of life. Considering the irreplaceable role of a vascularized flap in restoring the skull base, many scholars have presented multiple alternative resolutions regarding the lack of a whole HBF in secondary EEA surgery. These alternative vascularized flaps mainly originated from middle/inferior turbinate or pericranial tissues. In an anatomic dissection study, a pedicle middle turbinate flap was capable of covering a defect caused by EEA surgery (18). In clinical studies, some interesting results have been reported from a series of small cohort studies. Julián et al. proposed the utilization of a middle turbinate flap as a replacement when an HBF was not available (19). None of the patients suffered from postoperative CSF leakage in their cohort. Another study that enrolled more patients introduced by Carnevale et al. also evaluated the satisfactory effect of pedicled middle turbinate flaps in repeated EEA surgery (10). Furthermore, a pedicled inferior turbinate could also be employed as a reliable backup for HBF (9, 20). According to these studies, the pedicled middle/inferior turbinate flap is trustworthy when a whole HBF is absent. However, harvesting a pedicled middle/inferior turbinate flap will inevitably contribute to a more denuded nasal bone surface, which always leads to more severe nasal morbidity and poor sinonasal quality of life (18, 21). However, this issue has not attracted the full attention of neurosurgeons (13), and few neurosurgeons have specifically assessed alterations in nasal quality of life before and after EEA surgery. In addition, limited by the irregular shape and size of the turbinate, middle/inferior turbinate flaps are easily ruptured during the harvest process and might only fit a standard defect (22). Thus, when confronted with an expanded defect in secondary EEA surgery, pedicled middle/inferior turbinate flaps might not be sufficient. Apart from these pedicled nasal flaps, other scholars introduced a series of pedicled pericranial flaps, such as split-frontal pericranial flaps (23, 24) and temporoparietal fascia flaps (11), as substitutes for HBF in secondary EEA surgery. Although a watertight closure could be obtained by these pedicled pericranial flaps, they all need an extra incision to harvest the flap and a small bone window to rotate the flap into the nasal cavity, which inevitably causes extra injury for patients. In sum, for secondary EEA surgery, our strategy was not only a reliable solution but also prevented to bring extra damage to patients’ nasal quality of life.

In the present study, diabetes mellitus, artificial dura, craniopharyngioma and chordoma were detected to be independent risk factors for CSF complications in secondary EEA surgery.

According to Kiril E et al., diabetes mellitus patients are more vulnerable to certain infectious diseases, and the incidence of meningitis in diabetes patients is 2-fold higher than that in non-diabetes mellitus patients (25). Helene et al. declared that cell-mediated immunity might be severely disturbed in diabetics, especially when the normal function of polymorphonuclear leukocytes was obviously interfered with (26). Delamaire et al. also noted that neutrophil chemotaxis was weakened compared with controls after stimulation in both type I and type II diabetics (27). According to Sharipov et al. and Yu Jin et al., diabetes mellitus is one of the risk factors for nosocomial meningitis after endoscopic transsphenoidal surgery (28, 29). In the present study, for secondary EEA surgery, diabetes mellitus was an independent risk factor for CSF complications detected by the univariate and multivariate logistic regression and was more likely to induce meningitis and CSF leakage than pseudomeningocele according to multinomial logistic regression, which was consistent with previous studies.

Amanda et al. stated that clival chordoma was highly associated with postoperative CSF leakage in pediatric patients (30). According to a systematic review by Ethan et al., dural defects are always viably reconstructed by HBF, except those located in the clival region (17). Calvin et al. noted that dural defects located at the central skull base (sella and clival region) have an increased risk of CSF leakage compared to those of the anterior skull base (14). This phenomenon could be associated with multifactorial reasons. They proposed that an anterior skull base dura defect was always located at a horizontal plane, and the potential gap around the defect could be eliminated by counter pressure brought by the frontal lobe due to gravity. In contrast, regarding the clival and sella regions, a dural defect is always located at a vertical plane where there is a lack of tight counter pressure between the defect and the reconstructed material in fear of inducing more injuries to vital neural structures such as the optic nerve and brain stem. Shannon et al. also declared that lesions located in the posterior fossa are associated with a high rate of CSF leakage (16). In our cohort, craniopharyngioma and chordoma, which are both mainly located at the central skull base, were detected to be independent risk factors for postoperative CSF complications in secondary EEA surgery. Meanwhile, through multinomial logistic regression, the risk of each CSF complication was indiscriminately increased in patients suffering from chordoma. Moreover, based on our results, craniopharyngioma was more likely to induce meningitis, which might contribute to the prolonged stretching of brain tissue due to a lack of a clear interface between normal structures and lesions during secondary EEA surgery. Such injuries might subsequently induce astrocytic and microglial infiltration and related inflammatory responses (31, 32), which develop and persist during the perioperative period. Furthermore, a cystic craniopharyngioma could lead to postoperative chemical meningitis attributed to a leakage of cyst contents (33, 34).

According to previous literature, infection in situ, immune response, delayed healing, hemorrhage, bacterial and viral colonization are obviously associated with artificial duraplasty materials (35, 36). Other scholars also stated that artificial dura could trigger related chronic inflammation and the formation of granulation tissue due to its xenogeneic nature (37). Malliti et al. declared that dural defects reconstructed with synthetic dura are more susceptible to wound infection and CSF leakage. They also suggested that the utilization of synthetic dural grafts should be reserved except when autologous materials are unavailable (38). Consistently, the employment of artificial material was an independent risk factor for postoperative CSF complications, as confirmed by the univariate and multivariate logistic regression in the present study. Moreover, as detected by multinomial logistic regression, duraplasty with artificial material is more likely to increase the incidence of infection and CSF leakage than pseudomeningocele in secondary EEA surgery.

The major limitation of our study is its retrospective nature. Some patients’ first endonasal surgeries were not performed in our tertiary hospital. We could only speculate its first possible approaches and reconstruction strategies according to historical CT or MRI images. Thus, an accurate depiction about the approaches and reconstruction strategies performed in the first surgery is somewhat far-fetched, which might bring certain bias for our research. Meanwhile, patients who had previously experienced radiotherapy after initial surgery were excluded from our cohort, which might result in a certain population bias in our study. Thus, further prospective randomized controlled trials are needed to validate our results.

## Conclusion

Recycling an initial HBF along with a small piece of free autologous mucosa to reconstruct the skull base in secondary EEA surgery is a reasonable and effective method, especially for patients simultaneously suffering from diabetes mellitus and central skull base tumors.

## Data availability statement

The raw data supporting the conclusions of this article will be made available by the authors, without undue reservation.

## Ethics statement

The studies involving human participants were reviewed and approved by the Ethics Committee of the Seconds Affiliated Hospital of Air force Medical University of China. The patients/participants provided their written informed consent to participate in this study.

## Author contributions

Material preparation, data collection and analysis were performed by RW, GZ, and ZZ. The first draft of the manuscript was written by RW and all authors commented on previous versions of the manuscript. All authors contributed to the article and approved the submitted version.
